# Worldwide Dissemination of *bla*_KPC_ Gene by Novel Mobilization Platforms in *Pseudomonas aeruginosa*: A Systematic Review

**DOI:** 10.3390/antibiotics12040658

**Published:** 2023-03-28

**Authors:** Daniela Forero-Hurtado, Zayda Lorena Corredor-Rozo, Julián Santiago Ruiz-Castellanos, Ricaurte Alejandro Márquez-Ortiz, Deisy Abril, Natasha Vanegas, Gloria Inés Lafaurie, Leandro Chambrone, Javier Escobar-Pérez

**Affiliations:** 1 Bacterial Molecular Genetics Laboratory-LGMB, Universidad El Bosque, Ak. 9 #131a-02, Bogota 110121, Colombia; 2 The i3 Institute, Faculty of Science, University of Technology, Sydney, NSW 2007, Australia; 3 Unit of Basic Oral Investigations-UIBO, Universidad El Bosque, Bogota 110121, Colombia; 4 Evidence-Based Hub, Centro de Investigação Interdisciplinar Egas Moniz (CiiEM), Egas Moniz-Cooperativa de Ensino Superior, Caparica, 2829-511 Almada, Portugal; 5 Department of Periodontics, School of Dental Medicine, The University of Pennsylvania, Philadelphia, PA 19104, USA

**Keywords:** *Pseudomonas aeruginosa*, carbapenem resistance, KPC, Tn*4401*, NTE_KPC_, interactive map

## Abstract

The dissemination of *bla*_KPC_-harboring *Pseudomonas aeruginosa* (KPC-*Pa*) is considered a serious public health problem. This study provides an overview of the epidemiology of these isolates to try to elucidate novel mobilization platforms that could contribute to their worldwide spread. A systematic review in PubMed and EMBASE was performed to find articles published up to June 2022. In addition, a search algorithm using NCBI databases was developed to identify sequences that contain possible mobilization platforms. After that, the sequences were filtered and pair-aligned to describe the *bla*_KPC_ genetic environment. We found 691 KPC-*Pa* isolates belonging to 41 different sequence types and recovered from 14 countries. Although the *bla*_KPC_ gene is still mobilized by the transposon Tn*4401*, the non-Tn*4401* elements (NTE_KPC_) were the most frequent. Our analysis allowed us to identify 25 different NTE_KPC_, mainly belonging to the NTE_KPC_-I, and a new type (proposed as IVa) was also observed. This is the first systematic review that consolidates information about the behavior of the *bla*_KPC_ acquisition in *P. aeruginosa* and the genetic platforms implied in its successful worldwide spread. Our results show high NTE_KPC_ prevalence in *P. aeruginosa* and an accelerated dynamic of unrelated clones. All information collected in this review was used to build an interactive online map.

## 1. Introduction

Hospital-acquired infections are considered one of the biggest challenges to patient safety [1]. In Europe, it is estimated that 2,609,911 new cases of healthcare-associated infections (HCAIs) are reported each year [2], and on average, around 80,000 hospitalized patients have at least one HCAI on any given day [3,4]. In the United States (USA), it is estimated that every day 1 in 31 hospitalized patients carry an HCAI [5]. Infections caused by *Pseudomonas aeruginosa*, which are resistant to antibiotics, have become a serious public health problem, representing a risk factor for hospitalized patients, especially those in intensive care units (ICUs) [6]. In 2017, multidrug-resistant (MDR) *P. aeruginosa* caused an estimated 32,600 infections among hospitalized patients and approximately 2700 deaths in the USA [7].

According to the World Health Organization (WHO), *P. aeruginosa* has been classified within the “critical” category for the discovery of innovative treatments due to high rates of resistance [8,9]. The Centers for Disease Control and Prevention (CDC) in the USA reported that the rate of cases of hospital-onset MDR *P. aeruginosa* in 2020 increased by 32% compared to 2019 as a result of longer hospitalizations and bacterial infections associated with COVID-19 infections [10]; furthermore, in 2021, *P. aeruginosa* was the second most common microorganism isolated from adults in ICUs in hospitals in Colombia [11].

Although there is a wide range of antibiotics used to treat *P. aeruginosa* infections, such as ß-lactams, fluoroquinolones, aminoglycosides, and polymyxins [12], carbapenems are currently one of the most commonly used ß-lactam antibiotics for treating complicated *P. aeruginosa* infections [13,14]; however, the prevalence of carbapenem-resistant *P. aeruginosa* (CRPA) has increased rapidly, threatening the efficacy of these antibiotics and limiting the effective therapeutic options [6,15]. Due to the above, *P. aeruginosa* belongs to the “ESKAPE” list of pathogens of the Infectious Disease Society of America, which includes pathogens that represent a great threat to public health due to the ineffectiveness of multiple antibiotics against these bacteria [16].

*Klebsiella pneumoniae* carbapenemase (KPC) is a potent serine protease encoded by the *bla*_KPC_ gene that has a great clinical impact due to its high hydrolyzing activity of most β-lactams [17,18,19]. Since 1996, when KPC was first described in North Carolina (USA) in a *K. pneumoniae* isolate [20], most reports have been associated with this species and other *Enterobacteriaceae* [19,21]. However, in 2007, an isolate of *P. aeruginosa* with a high level of resistance to carbapenems harboring *bla*_KPC_ was reported in Medellin, Colombia [22]; since then, there have been additional reports of such isolates, mainly in the Americas and Asian countries [18].

Initial dissemination of *bla*_KPC_ was exclusively associated with the Tn*3*-family Tn*4401* transposon, which has a size of 10 Kb and very active transposition without target site specificity [23]. However, *bla*_KPC_-positive and Tn*4401*-negative plasmids were later found to harbor different transposases that were related to the mobilization of this gene. These new structures were denominated as NTE_KPC_ (non-Tn*4401* elements) [24], and it has been presumed that they could have facilitated *bla*_KPC_ mobilization due to their smaller size and higher transposition frequencies [25]. The first *bla*_KPC_-plasmids described in *P. aeruginosa* were pCOL-1 and pPA-2, containing the *bla*_KPC_ gene within the Tn*4401* transposon and NTE_KPC_-II element, respectively [26,27].

The worldwide dissemination of *bla*_KPC_-harboring *P. aeruginosa* (KPC-*Pa*) could be associated with their ability to adapt through genomic plasticity [13,28] and the success of different plasmids and transposons, particularly the Tn*4401* transposon (mainly in its *b* isoform), the most frequently associated with *bla*_KPC_ mobilization [29]. However, the high incidence of NTE_KPC_-flanked *bla*_KPC_ [30,31] suggests they may play an important role in the diffusion of resistance determinants in hospital environments.

Several studies have examined the impact of resistant patterns in Gram-negative bacilli and MDR *P. aeruginosa* strains, but few studies focus on the analysis of the new mobile genetic elements (MGE) associated with resistance genes in CRPA. The discovery of *bla*_KPC_ acquisition by *P. aeruginosa* amid an explosion of mass genome sequencing techniques has given us an excellent opportunity to closely track emerging MGEs to better understand their worldwide mobilization dynamics. Thus, the aim of this systematic review was to identify the genetic elements associated with *bla*_KPC_ in CRPA and disentangle the possible mechanisms responsible for the worldwide dissemination of these strains.

## 2. Results

### 2.1. Search Results

**Figure 1:**
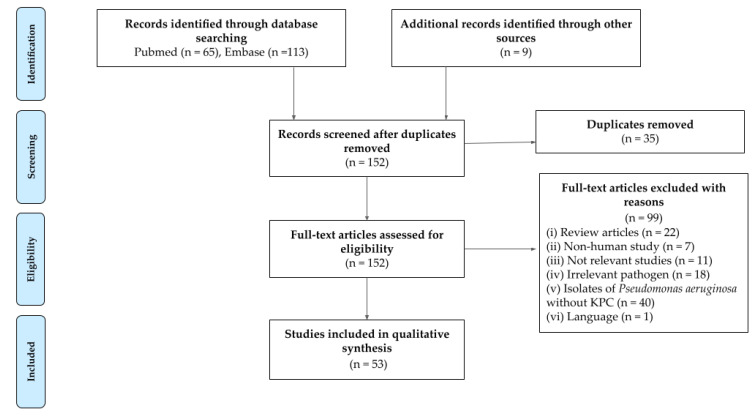
Flow diagram of study selection.

A systematic literature search resulted in a total of 178 articles with publication dates ranging from January 2007 to June 2022. Nine additional articles were obtained via a manual search. After removing duplicates, 152 articles were eligible for full-text review. Finally, 53 studies were included in the systematic review after evaluating the inclusion/exclusion criteria (Figure 1). The detailed characteristics of the articles are presented in Table 1.

The 53 studies included in this review were conducted in American, European, and Asian countries. China (14 studies) [14,15,17,19,32,33,34,35,36,37,38,39,40,41], Brazil (10 studies) [21,42,43,44,45,46,47,48,49,50], and Colombia (9 studies) [22,26,27,28,29,51,52,53,54] were the countries with the most reported studies, followed by Puerto Rico (4 studies) [55,56,57,58], Chile (2 studies) [59,60], Argentina (2 studies) [61,62], Vietnam (1 study) [63], Trinidad and Tobago (1 study) [64], Nepal (1 study) [65], India (1 study) [66], Germany (1 study) [18], Spain (1 study) [6], and the USA (1 study) [67]. From the remaining five papers, only three studies were conducted in an unclear location [68,69,70], and two studies worked with isolates at a global level (Table 1) [71,72]. These results demonstrate the extent of the global spread of *bla*_KPC_-positive *P. aeruginosa* strains.

### 2.2. Geographical Distribution and Genetic Relationship of bla_KPC_-Harboring P. aeruginosa Isolates

From the 53 evaluated studies, 704 KPC-*Pa* were found; however, thirteen isolates were reported in more than one article; hence the final value of KPC-*Pa* identified in the review was 691. The *bla*_KPC-2_ was the most frequent variant present in 567 (81.9%) isolates, followed by *bla*_KPC-5_, *bla*_KPC-33_, and *bla*_KPC-90_ in two, one, and one isolates, respectively. In the remaining 120 isolates (17.3%), the *bla*_KPC_ variant was not specified. Most isolates were reported in China (362; 52.3%), followed by Puerto Rico (121; 17.5%), Colombia (81; 11.7%), Chile (43; 6.2%), Argentina (33; 4.7%), Brazil (29; 4.2%), Vietnam (7; 1.0%), Nepal (4; 0.5%), Guatemala (3; 0.4%), India (2; 0.2%), Spain (2; 0.2%), the USA (1; 0.1%), Germany (1; 0.1%), and Trinidad and Tobago (1; 0.1%).

**Figure 2:**
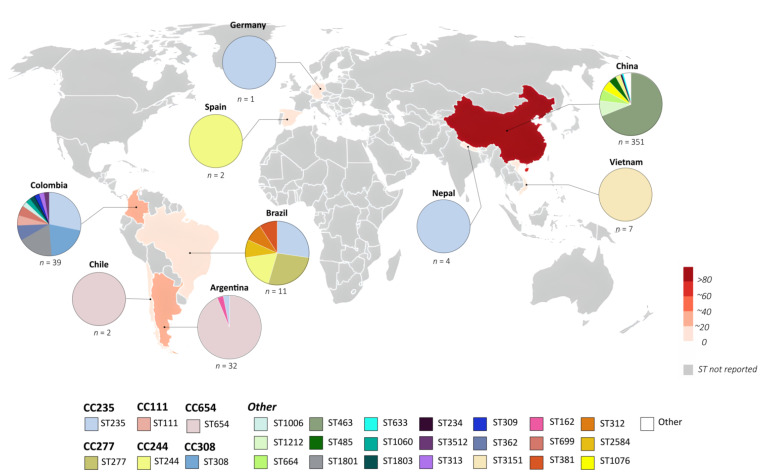
Geographic distribution of the *bla*_KPC_-harboring *Pseudomonas aeruginosa* isolates with known sequence types. The color shading represents the total number of isolates with known sequence types. Pie charts refer to the proportion of representative sequence *bla*_KPC_-harboring types found per country. Clonal complexes (CC) were assigned according to Del Barrio-Tofiño et al. in 2020 [73].

Regarding the genetic relation of the isolates, forty-one different sequence types (STs) were identified in 449 KPC-*Pa* in nine different countries (Figure 2); the most predominant ST was ST463, to which 242 (53.9%) isolates belonged; other relevant STs were ST654, ST1212, ST664, and ST235 found in 32 (7.1%), 28 (6.2%), 21 (4.6%), and 20 (4.4%) isolates, respectively. In China, ST463 was the most frequent (68.9%) and exclusive to this country. Likewise, for ST654, 93.7% of the reports were part of the same study that evaluated isolates located in different hospitals in Argentina [61]. Although the pandemic clone ST235 does not have a high frequency, it has been described in at least five different countries. Furthermore, ST209 and ST274 reported in two studies conducted in Zhejiang, China [15,33], were the only two STs with single locus variations (SLV). The great diversity of STs identified reveals an alarming increase of unrelated clones that have acquired the *bla*_KPC_ gene; in addition, there is a predominance of specific STs among populations with different geographical locations, which may have significant public healthcare implications.

**Table 1:** Characteristics of the fifty-three studies included in the systematic review.

First Author	Year	Continent	Country	Collection Date ^1^	Isolates (*n* = 704) ^2^	KPC Variant ^3^	Sequence Types	Ref
Villegas	2007	South America	Colombia	2006	3 (0.4)	KPC (2/3), KPC-2 (1/3)	NS (3/3)	[22]
Naas *	2008	NS	NS	NS	1 (0.1)	KPC-2 (1/1)	NS (1/1)	[68]
Wolter	2009	Middle America	Puerto Rico	2006–2007	25 (3.5)	KPC (18/25), KPC-2 (6/25), KPC-5 (1/25)	NS (25/25)	[55]
Wolter *	2009	Middle America	Puerto Rico	2006	2 (0.2)	KPC-2 (1/2), KPC-5 (1/2)	NS (2/2)	[56]
Akpaka	2009	South America	Trinidad and Tobago	NS	1 (0.1)	KPC-2 (1/1)	NS (1/1)	[64]
Poirel *	2010	North America	USA	2009	1 (0.1)	KPC-2 (1/1)	NS (1/1)	[67]
Ge *	2011	Asia	China	2009	3 (0.4)	KPC-2 (3/3)	ST463 (3/3)	[32]
Cuzon *	2011	South America	Colombia	2006–2010	10 (1.4)	KPC-2 (10/10)	ST308 (6/10), ST235 (2/10), ST1006 (1/10), ST1060 (1/10)	[26]
Robledo	2011	Middle America	Puerto Rico	2009	89 (12.6)	KPC (89/89)	NS (89/89)	[57]
Martínez *	2012	Middle America	Puerto Rico	2009	1 (0.1)	KPC-2 (1/1)	NS (1/1)	[58]
Jácome	2012	South America	Brazil	2010	2 (0.2)	KPC-2 (2/2)	NS (2/2)	[42]
Pasteran *	2012	South America	Argentina	2006–2011	30 (4.2)	KPC-2 (30/30)	ST654 (29/30), ST162 (1/30)	[61]
Correa *	2012	South America	Colombia	2010	1 (0.1)	KPC-2 (1/1)	ST111 (1/1)	[51]
Roth *	2013	NS	NS	NS	1 (0.1)	KPC-2 (1/1)	NS (1/1)	[69]
Naas *	2013	South America	Colombia	NS	2 (0.2)	KPC-2 (2/2)	ST308 (1/2), ST1006 (1/2)	[27]
Buelvas	2013	South America	Colombia	2008	1 (0.1)	KPC-2 (1/1)	NS (1/1)	[52]
Vanegas	2014	South America	Colombia	2012–2014	25 (3.5)	KPC-2 (25/25)	ST1801 (7/25), ST235 (5/25), ST362 (3/25), ST111 (1/25), ST1803 (1/25), NS (8/25)	[53]
Cavalcanti	2015	South America	Brazil	2008–2010	3 (0.4)	KPC-2 (3/3)	ST235 (2/3), ST244 (1/3)	[43]
Hu *	2015	Asia	China	2013	39 (5.5)	KPC-2 (39/39)	ST463 (31/39), ST1076 (2/39), ST1755 (1/39), ST850 (1/39), ST357 (1/39), ST836 (1/31), ST209 (1/39), ST244 (1/39)	[33]
Paul *	2015	Asia	India	2012–2013	2 (0.2)	KPC-2 (2/2)	NS (2/2)	[66]
Dai *	2016	Asia	China	2013	1 (0.1)	KPC-2 (1/1)	NS (1/1)	[17]
Kazmierczak	2016	America/Asia	Global data	2012–2014	29 (4.1)	KPC-2 (29/29)	NS (29/29)	[71]
Galetti *	2016	South America	Brazil	2011	1 (0.1)	KPC-2 (1/1)	ST244 (1/1)	[44]
Hagemann *	2018	Europe	Germany	NS	1 (0.1)	KPC-2 (1/1)	ST235 (1/1)	[18]
de Oliveira Santos *	2018	South America	Brazil	2014	1 (0.1)	KPC-2 (1/1)	ST2584 (1/1)	[21]
Shi *	2018	Asia	China	2016	1 (0.1)	KPC-2 (1/1)	NS (1/1)	[34]
de Paula-Petroli	2018	South America	Brazil	2008	1 (0.1)	KPC-2 (1/1)	ST235 (1/1)	[45]
Galetti *	2019	South America	Brazil	2011	1 (0.1)	KPC-2 (1/1)	ST381 (1/1)	[46]
Hu *	2019	Asia	China	2010	1 (0.1)	KPC-2 (1/1)	ST463 (1/1)	[35]
Pacheco	2019	South America	Colombia	2017	5 (0.7)	KPC-2 (5/5)	NS (5/5)	[54]
Abril *	2019	South America	Colombia	2014–2016	4 (0.5)	KPC-2 (4/4)	ST235 (4/4)	[29]
Li *	2020	Asia	China	2018	21 (2.9)	KPC-2 (21/21)	ST664 (21/21)	[19]
Pérez-Vázquez	2020	Europe	Spain	2016	2 (0.2)	KPC-2 (2/2)	ST244 (2/2)	[6]
Tartari *	2021	South America	Brazil	2018	1 (0.1)	KPC-2 (1/1)	ST312 (1/1)	[49]
Cai *	2021	Asia	China	2019	4 (0.5)	KPC-2 (4/4)	ST463 (4/4)	[14]
Wozniak *	2021	South America	Chile	2015	2 (0.2)	KPC-2 (2/2)	ST654 (2/2)	[60]
Rada *	2021	South America	Colombia	2013–2015	12 (1.7)	KPC-2 (12/12)	ST308 (2/12), ST699 (2/12), ST309 (1/12), ST313 (1/12), ST3512 (1/12), NS (5/12)	[28]
Hu *	2021	Asia	China	2007–2018	105 (14.9)	KPC-2 (105/105)	ST463 (71/105), ST1212 (13/105), ST1076 (10/105), ST9 (1/105), ST209 (1/105), ST244 (1/1015), ST274 (1/105), ST277 (1/105), ST360 (1/105), ST377 (1/105), ST836 (1/105), ST1642 (1/105) ST2235 (1/105), NS (1/105)	[15]
Tran	2021	Asia	Vietnam	2011–2013	7 (0.9)	KPC-2 (7/7)	ST3151 (7/7)	[63]
Souza	2021	South America	Brazil	2015–2016	3 (0.4)	KPC-2 (3/3)	NS (3/3)	[48]
Costa-Júnior	2021	South America	Brazil	2018–2019	11 (1.5)	KPC (11/11)	NS (11/11)	[47]
Hu *	2021	Asia	China	2014–2019	16 (2.2)	KPC-2 (16/16)	ST463 (7/16), ST1076 (3/16), ST1212 (3/16), ST633 (2/16), NS (1/16)	[37]
Yuan *	2021	Asia	China	2015	1 (0.1)	KPC-2 (1/1)	NS (1/1)	[39]
Zhu *	2021	Asia	China	2017–2018	151 (21.4)	KPC-2 (151/151)	ST463 (107/151), ST485 (14/151), ST1212 (12/151), ST244 (7/151), ST234 (2/151), ST1076 (2/151), ST606 (1/151), ST1631 (1/151), ST3217 (1/151), NS (4/151)	[40]
Hu *	2021	Asia	China	2019–2020	24 (3.4)	KPC-2 (23/24), KPC-33 (1/24)	ST463 (23/24), ST1076 (1/24)	[36]
Costa	2021	South America	Chile	2015–2018	19 (2.7)	KPC-2 (19/19)	NS (19/19)	[59]
Wang *	2021	Asia	China	2017	1 (0.1)	KPC-2 (1/1)	NS (1/1)	[38]
Cardinal	2021	South America	Global data	2017–2019	24 (3.4)	KPC-2 (24/24)	NS (24/24)	[72]
Takahashi *	2021	Asia	Nepal	2018–2020	4 (0.5)	KPC-2 (4/4)	ST235 (4/4)	[65]
Cejas *	2022	South America	Argentina	2008 and 2018	2 (0.2)	KPC-2 (2/2)	ST654 (1/2), ST235 (1/2)	[62]
Tu *	2022	Asia	China	2021	1 (0.1)	KPC-90 (1/1)	ST463 (1/1)	[41]
Li *	2022	NS	NS	NS	2 (0.2)	KPC-2 (2/2)	ST463 (2/2)	[70]
Silveira *	2022	South America	Brazil	2020	3 (0.4)	KPC-2 (3/3)	ST277 (3/3)	[50]

^1^ Corresponds to the date on which the clinical isolation was obtained. ^2^ Data are n (%) of isolates. ^3^ Proportion of KPC variants by the number of isolates reported in the article. * Article reporting the genetic location (plasmid or chromosome) or *bla*_KPC_ environment (Tn*4401* or NTE_KPC_). Abbreviations: NS, information not specified in the original article.

### 2.3. Genetic Platforms Mobilizing bla_KPC_ Gene in P. aeruginosa

Out of all the isolates, only 234 KPC-*Pa* (36 studies) described the genetic location (plasmid or chromosome) and/or genetic structures surrounding *bla*_KPC_ (transposons, NTE_KPC,_ or insertion sequences). With regard to their genetic location, 199 (84.6%) of these isolates harbored *bla*_KPC_ within a plasmid structure, 6 (2.5%) isolates contained the gene in the chromosome, and 1 isolate presented a report of *bla*_KPC_ in the plasmid and chromosome. The remaining 28 (11.9%) KPC-*Pa* isolates did not emphasize the genetic location but did report the genetic environment adjacent to the *bla*_KPC_.

Of the 200 reports of *bla*_KPC_ encoded within a plasmid, 52 (26.0%) reported the gene as part of an NTE_KPC_ element, 44 (22.0%) in a Tn*4401* transposon variant, and 1 isolate (0.5%) reported *bla*_KPC_ within an integron-like genetic structure [66]. KPC-associated plasmids varied widely in size, from small plasmids of less than 4 Kbp to mega plasmids greater than 400 Kbp [19,44]. Furthermore, it was possible to identify seven different incompatibility groups: IncP [50], IncP-3-like (IncA/C) [19], IncU [27,49], IncP-6 [17,27,38], IncF-like [66], IncQ1 [21], and IncHI1 [18]. According to the information collected, only 24 *bla*_KPC_-carrying *P. aeruginosa* plasmids were fully sequenced and are available both in the NCBI public database and the published literature (Table 2).

In the case of the seven reports harboring *bla*_KPC_ on the chromosome, five (71.4%) of these reports were associated with an NTE_KPC_ element and the other two (28.5%) with a Tn*4401*-like structure. Although the remaining 28 isolates did not specify the location of this gene on the plasmid or chromosome, 20 (71.4%) of them were associated with NTE_KPC_ and 8 (28.5%) with Tn*4401.*

### 2.4. In Silico Assessment of the bla_KPC_ Genetic Environment on P. aeruginosa Isolates

**Figure 3:**
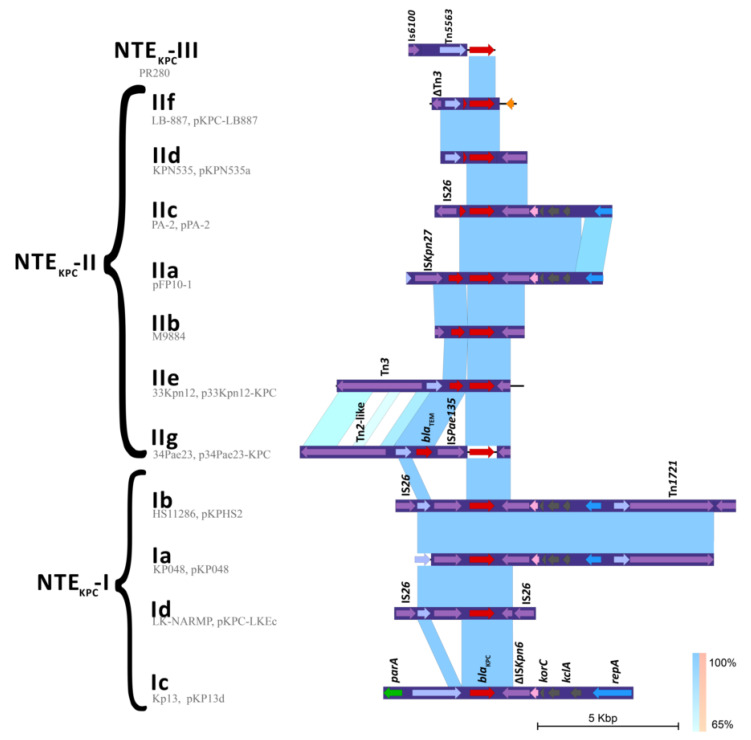
Schematic comparison between the different subgroups of NTE_KPC_s (non-Tn*4401* elements). This image is an update of the information presented by Chen et al. in 2014 [24] about NTE_KPC_ elements. Within the known NTE_KPC_, three groups are recognized: I, II, and III. The shaded area between the sequences delimits the alignment regions with a percentage identity of ≥65%. The red, purple, lilac, blue, and gray arrows indicate the *bla*_KPC_ gene, transposases, resolvases, replicative proteins, and other open-reading frames, respectively. The graph has a scale line of 5000 bp. The NTE_KPC_ GenBank accession numbers can be consulted in Supplementary Material Table S1.

Due to the high presence of possible NTE_KPC_ in these isolates, an analysis to characterize the *bla*_KPC_ genetic environment was performed. The genetic environments of the isolates that were collected from the NCBI nucleotide database were compared to establish their relationship. Since Chen et al. coined the term NTE_KPC_ in 2014 for non-Tn*4401* elements adjacent to the *bla*_KPC_ found mainly in *K. pneumoniae* [24], three types (I, II, and III) and several subtypes have been reported. Currently, when a new structure is found, the assignment of type and subtype relies on the author. This has generated some problems such as the duplication of names in different structures; for instance, two of these elements that were reported in different species were both named as NTE_KPC_-IIe despite presenting structural differences between them; specifically, the *bla*_TEM_ region that both environments presented is not shared. Although both present a *tnpA* of the Tn*3* family upstream of the resolvase, they do not seem to have similarities in most of their structure. In this sense, to unify the nomenclature, we have renamed some structures according to their order of appearance. Thus, the NTE_KPC_-IIe that was reported by Campana [74] was renamed NTE_KPC_-IIf, and the structure reported by Abril et al. (NCBI accession number CP095773.1) as NTE_KPC_-IIf has received the name NTE_KPC_-IIg (Figure 3).

**Figure 4:**
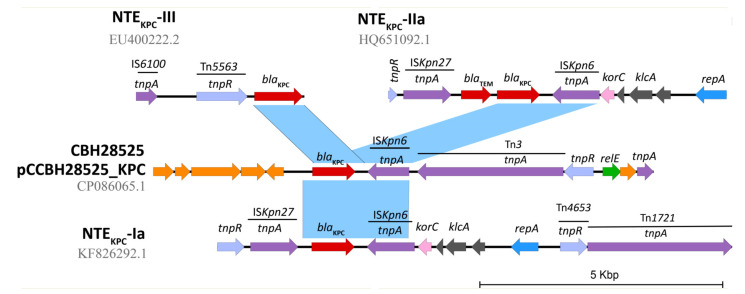
Schematic comparison of the *bla*_KPC_ surroundings located in the plasmid pCCBH2825-KPC and the NTE_KPC_ (non-Tn*4401* elements) subtypes I, II, and III. As it is observed, the *bla*_KPC_ surroundings in the plasmid pCCBH2825-KPC harbor different genes with respect to the other three NTE_KPC_ types previously described. To highlight, the presence of the *tnpA* gene downstream that encodes for a Tn*3*-related transposase is probably associated with the *bla*_KPC_ mobilization. The shaded area between the sequences delimits the alignment regions with a percentage identity of ≥90%. The red, purple, lilac, blue, and gray arrows indicate the *bla*_KPC_ gene, transposases, resolvases, replicative proteins, and other open-reading frames, respectively. The graph has a scale line of 5000 bp.

From our results, 25 different and novel environments were found in GenBank, which have not previously been reported as NTE_KPC_. Supplementary material (Table S1) shows the list of isolates harboring novel NTE_KPC_ structures. Among these, seventeen (68.0%) were reported as belonging to subtype I, six to subtype II (24.0%), one to subtype III (4.0%), and one that could not be classified within the three existing subtypes of NTE_KPC_ (4.0%) (pCCBH8525_KPC). The *bla*_KPC_ gene found within the plasmid pCCBH28525_KPC (Table 2) did not present any of the surrounding characteristic genes of the NTE_KPC_ subtypes already described: IS*Kpn27* in the case of NTE_KPC_-I; *bla*_TEM_ for NTE_KPC_-II; and IS*6100*/Tn*5563* resolvase for NTE_KPC_-III [24]. Instead, the genes encoding hypothetical proteins were located in the upstream region, and genes related to transposition, such as IS*Kpn6*, *tnpA* (encoding a Tn*3*-related transposase), and *tnpR*, were found downstream (Figure 4). For this reason, we considered that this structure corresponds to a new type, namely NTE_KPC_-IVa (CP086065.1). It should be clarified that in the present study, the frequency of these novel genetic environments was not evaluated as the intention was to report the differences among novel mobilization platforms found in *P. aeruginosa* compared to those previously reported [24].

**Figure 5:**
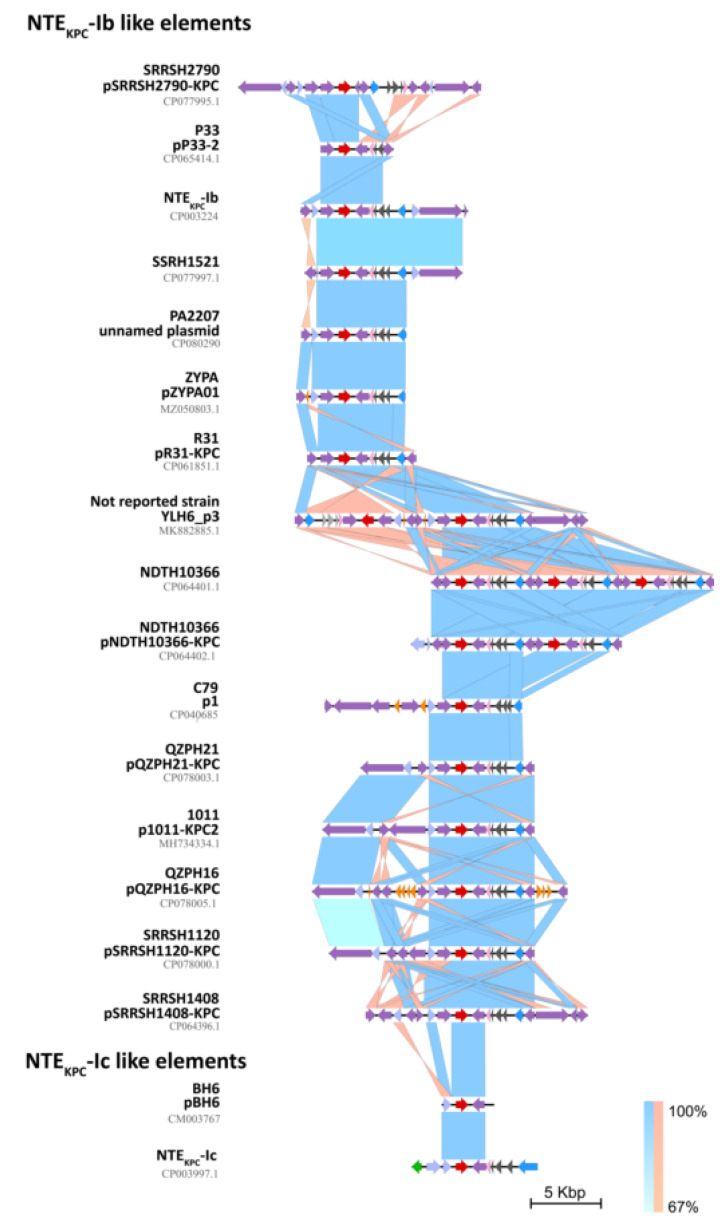
Comparison of the compiled environments that were considered as novel NTE_KPC_-I elements. Two different subgroups could be identified; NTE_KPC_-Ib-like elements and NTE_KPC_-Ic-like elements. The shaded area between the sequences delimits the alignment regions with a percentage identity of ≥ 67%. The red, purple, lilac, blue, and gray arrows indicate the *bla*_KPC_ gene, transposases, resolvases, replicative proteins, and other open-reading frames, respectively. The graph has a scale line of 5000 bp.

On the other hand, a large part of the 25 novel environments that were classified as NTE_KPC_-I was subclassified as NTE_KPC_-Ib-like elements as they contain the highly conserved region made up of an incomplete version of the IS*Kpn6*, *korC*, *klcA*, and *repA* genes downstream to *bla*_KPC_. In the upstream region, they contained IS*Kpn27* and, in many cases, a resolvase (*tnpR*). They also presented different transposable elements, such as the *tnpR* of Tn*4653*, Tn*1721*, and Tn*2* transposases, or different copies of the IS*26*, depending on the isolate (Figure 5).

NTE_KPC_-I was the only type found to be chromosomally located with subtle structure variations. For instance, the region surrounding chromosomal *bla*_KPC_ in isolate SRRSH1521 (ST244) (CP077997.1) showed 97.2% homology to a region covering 87% of the NTE_KPC_-Ib gene, with the major difference being the insertion of an IS*6100* copy (instead of IS*26*) into the *tnpR* gene. In addition, an NTE_KPC_-Ic-like element was also identified in the isolate BH6 (CM003767.1) but with some deletions upstream and downstream of the *bla*_KPC_ gene (Figure 5).

**Figure 6:**
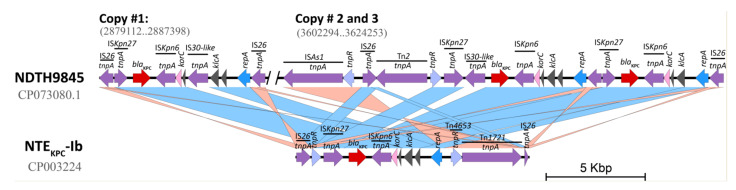
Organization of the region harboring three copies of NTE_KPC_-Ib in the isolate NDTH9845. The shaded area between the sequences delimits the alignment regions with a percentage identity of 100%. The red, purple, lilac, blue, and gray arrows indicate the *bla*_KPC_ gene, transposases, resolvases, replicative proteins, and other open-reading frames, respectively. The graph has a scale line of 5000 bp.

An interesting and complex chromosomal NTE_KPC_ structure was characterized for isolate NDTH9845, where three copies of *bla*_KPC_ were mobilized through very similar NTE_KPC_-Ib platforms. Some differences among these three platforms were the insertion of different IS*30* copies and the Tn*2*-like *tnpA* gene (Figure 6).

**Figure 7:**
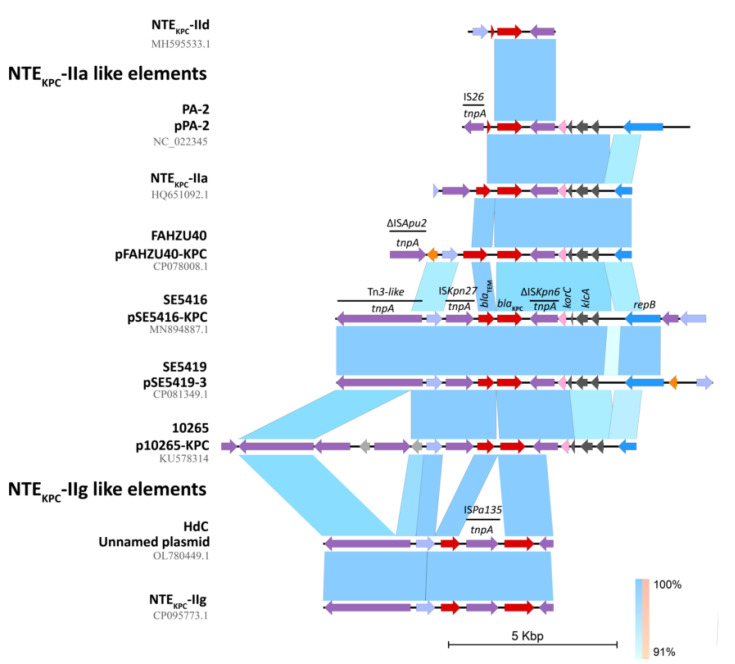
Comparison of novel NTE_KPC_-II elements compiled in this study. The shaded area between the sequences delimits the alignment regions with a percentage identity of ≥91%. The red, purple, lilac, blue, and gray arrows indicate the *bla*_KPC_ gene, transposases, resolvases, replicative proteins, and other open-reading frames, respectively. The graph has a scale line of 5000 bp.

Among the six isolates that contained structures related to NTE_KPC_-II, five were considered NTE_KPC_-IIa-like elements, presenting some structural differences, such as the deletion of IS*Kpn27* (i.e., isolate pFAHZU40-KPC); insertion of transposable elements, such as the IS*26*, IS*Apu2*, IS*Apu1*, Tn*3*, and Tn*2*-like transposases; or changes in the intergenic regions (Figure 7). Interestingly, the latest NTE_KPC_-II described corresponds to the recently described NTE_KPC_-IIg, which was also found in different clones recovered from Colombia and Argentina, which are two distant countries, suggesting a wide dissemination of this platform (access through CP095773.1 and OL780449.1, respectively).

Finally, only one isolate presented an NTE_KPC_-III environment. This case is particular as it harbors IS*6100* (a hallmark of the NTE_KPC_-III) in the form of a chimera with a previously unreported transposase. This transposase shows similarities with those of the Tn*3* or IS*481* family. In addition, genes normally found in other NTE_KPC_ subtypes, such as types I or II, are found downstream and correspond to *korC*, *klcA*, and *repB.*

### 2.5. Interactive Online Map Construction

The data from this systematic review show only an approximation of the current landscape of *bla*_KPC_-harboring CRPA. The SARS-CoV-2 pandemic taught us the importance of generating interactive tools to collect worldwide information about infectious pathogens in real time. Based on this, we summarized the valuable information presented here on *bla*_KPC_ acquisition and spread in *P. aeruginosa* in a world online map tool, which can be consulted at the following link: https://maphub.net/LGMB/KPC-Pseudomonas-aeruginosa-LGMB. The information contained in this map will be updated periodically with new genomic reports of KPC-*Pa* isolates.

## 3. Discussion

This study systematically reviewed fifty-three studies and provides a first insight into the impacts of genetic platforms on the dissemination of *bla*_KPC_ in *P. aeruginosa* and shows an overview of the global epidemiology of *bla*_KPC_-harboring CRPA. Since the first report of KPC-*Pa* in 2007 [22], the number of these *bla*_KPC_-harboring isolates has been increasing, especially in Asia and South America; specifically, China, Brazil, Colombia, and Puerto Rico are the countries with the largest number of reports on the subject. According to the Antimicrobial Testing Leadership and Surveillance program [75], the rate of resistance to carbapenems (reported in 2018) in *P. aeruginosa* clinical strains was highest in the Middle East, followed by South America, Europe, and North America [13], suggesting that KPC may potentially be contributing to the increase and spread of these patterns of resistance in conjunction with other carbapenemases (i.e., VIM) and resistance mechanisms such as the repression of the OprD porin and the overexpression of efflux pumps [15,28,76].

The WHO classified CRPA as a critical priority on the list of the most dangerous pathogens that need the development of new antimicrobial drugs [77]. Even though *bla*_KPC_ is still most prevalent in *K. pneumoniae* [18], our findings suggest it may also play a role in the ability of *P. aeruginosa* to exhibit genome plasticity and adapt to different conditions across 14 countries [13].

The current systematic review indicated that KPC-2 remains the most successful KPC variant worldwide, followed by KPC-5, which has only been reported in Puerto Rico. Recently, KPC-33 and KPC-90 variants have also been identified in Zhejiang, China, both belonging to ST463 [36,41]. Although all isolates have reported resistance against carbapenem antibiotics, some variants, such as KPC-90, have already shown to be resistant to more advanced combinations of antibiotics, such as ceftazidime–avibactam (CZA), a treatment that has demonstrated high efficacy against *bla*_KPC-2_-harboring CRPA strains [41].

In addition, there is an isolate that was recently identified by us (not yet published), which was not part of the review. This isolate apparently corresponds to the first report of *bla*_KPC-3_ in *P. aeruginosa*, is associated with Tn*4401b*, and was found in the pandemic high-risk clone ST111. The appearance of the second most widespread KPC-3 variant [78] within a highly active transposon mainly responsible for KPC dissemination in carbapenem-resistant *Enterobacteriaceae* [24] opens a possible new expansion route for this enzyme. Furthermore, since the completion of this review, there have been reports of KPC-3 variants, such as KPC-31, which are also resistant to CZA and were found in a high-risk clone ST235 [79]. These findings underline the importance of continued surveillance.

We have identified more than forty-one different STs, of which only two (ST209, ST274) presented an SLV. This finding demonstrates a wide heterogeneous distribution of non-related clones. These SLVs will diversify over time, generating new variants, likely including double locus variants (DLV) and triple locus variants (TLV) [80]. Additionally, seven out of ten high-risk clones reported for *P. aeruginosa* (ST111, ST244, ST235, ST277, ST308, ST357, and ST654) were identified [13], and the ST that showed the greatest spread was ST235, following reports in Argentina, Brazil, Colombia, Germany, and Nepal. Our results are consistent with previous studies that describe the population structure of *P. aeruginosa* as non-clonal and epidemic [81]. However, it was observed that the ST463 reports correspond mostly to hospitals located in East China, which could represent endemicity for this clone [36]. Likewise, it has been shown that the high-risk clone ST654 is playing a key role in the spread of KPC-*Pa* in Argentina [61] as opposed to different clones being responsible for its spread in Colombia.

The global expansion of carbapenem resistance in Gram-negative bacteria can be attributed to horizontal gene transfer mediated by active transposons and multiple plasmids [36]. This has allowed enzymes, such as KPC, to be more easily transferred and become endemic in various places, such as the USA, Argentina, Brazil, Colombia, Eastern China, Greece, Israel, and Italy [24,36]. The worldwide distribution of *bla*_KPC_ in *K. pneumoniae* has been attributed mainly to two factors: (i) the global dissemination of the clonal group CG258 and (ii) the localization of *bla*_KPC_ in the Tn*4401* transposon variants harbored in different plasmids [60,82]. Nonetheless, for *P. aeruginosa*, we observed an alarming increase in unrelated clones and the localization of *bla*_KPC_ within NTE_KPC_ structures. In this review, we continue to observe a high incidence of Tn*4401* elements, among which their isoform *b* stands out, but Tn*4401a* is also reported. Likewise, it has been shown that the wide circulation of plasmids carrying *bla*_KPC_ has resulted in an increase in resistant isolates reported in hospitals. Up to now, twenty-four *bla*_KPC_-carrying plasmids from *P. aeruginosa* have been available in the NCBI public databases and the published literature. It would be interesting to understand the possible associations between plasmids and mobilization platforms, which may be the subject of future studies.

On the other hand, the *bla*_KPC_ gene has been reported within these conventional genetic elements in several bacterial species, including *P. aeruginosa* [24]. The presence of NTE_KPC_ in the KPC-*Pa* isolates identified in this review shows that these elements may be contributing to the dissemination of KPC. In addition, our in silico analysis shows a great diversity of NTEs, with NTE_KPC_-I being the most prevalent. However, in some cases, the NTE_KPC_-Ib-like elements were present as several copies, resulting in chimeras with various copies of *bla*_KPC_. There was a case (chromosome of NDTH7329 strain) in which six identical copies of the same genetic environment were present (accession number CP078006.1). The presence of transposases, such as IS*26* and Tn*3* family transposases, including Tn*3* itself, Tn*2* or Tn*As1*, and Tn*A2*, may suggest that the mobilization of the NTE_KPC_-I elements through the Russian doll model in which transposable elements are mobilized through adjacent transposable elements [83]. The frequency of IS*26* in the isolates evaluated by this study is a particularly interesting result because IS26 can be mobilized in various ways; in fact, it is believed that it could mobilize neighboring genes without the action of another transposase [84].

It is noteworthy that there were isolates in which the same mobilization platform was present, as in the case of the mobilization platform found in the SRRSH1521 (CP077997.1) chromosome, where: (i) theYLH6_p3 plasmid mobilization platform was also present in the SRRH1042 plasmid, and (ii) the mobilization elements in pZYPA01 were also present in pP23-KPC. This suggests that these elements are able to transfer between plasmids, at least within the Chinese territory where these cases were reported. Despite the high incidence in NTE_KPC_ reports, it is still not clear whether these genetic structures can be mobilized independently or whether their successful dissemination is due to the involvement of various plasmids, so further analysis of these platforms is warranted. The NTE_KPC_-IIe is the only element of this kind that has shown defined inverted repeats (IRs) in addition to presenting direct repeats as an indication of its transposition [85].

The present systematic review encountered the following limitations: Due to the large amount of information related to CRPA strains, our search strategy was focused on mobilization platforms; this could exclude relevant information from studies with a different approach. In addition, although duplicate isolates were not considered for analysis in this study, there are studies that reported multiple isolates possibly associated with epidemic outbreaks or surveillance studies, which causes an over-representation in some of the data presented in the review. However, as they are independent isolates, it was decided to evaluate all the information to avoid losing relevant data, such as STs. Finally, most of the evaluated data in this review required bioinformatics information that many of the articles do not present.

## 4. Materials and Methods

### 4.1. Search Strategy

A systematic review was conducted following the published guidelines for the development of systematic reviews [86]. Online searches were performed through the PubMed and EMBASE databases for articles published up to June 2022, without geographic location restriction. A combination of keywords, controlled vocabulary (MeSH/Emtree terms), and Boolean operators (“AND” and “OR”) were used during the search. The initial search strategy was designed using a fitted PICO (population, intervention, comparison, outcome) model and combining terms related to the pathogen (“*Pseudomonas aeruginosa*”), carbapenem antibiotics (“carbapenem”, “doripenem”, “ertapenem”, “imipenem”), the resistance gene *bla*_KPC_ (“beta-lactamase KPC”, “*bla*_KPC_”, “KPC”), and some dissemination platforms or MGE (“dissemination”, “transposon”, “plasmids”, “DNA transposable elements”) that could be associated with the spread of these strains worldwide. Supplementary Material Table S2 shows the full search strategies for each database. Additionally, via hand-searching, we complemented the search to include relevant articles that were missed during indexing.

The initial protocol was registered in the Prospective International Registry of Systematic Reviews (PROSPERO) of the National Institute of Health Research (Registration code: CRD42022320686).

### 4.2. Inclusion and Exclusion Criteria

Full-text retrieved items were screened to determine their eligibility according to the predefined selection criteria. Based on the objective, all the observational studies related to KPC-*Pa* isolates from clinical samples of patients with resistance to at least one type of carbapenem antibiotic were considered for inclusion in the systematic review. Furthermore, studies that reported dissemination platforms or MGE related to *bla*_KPC_ and/or studies that reported STs associated with the isolates were included. Furthermore, we included some articles that did not describe the dissemination platforms but were considered relevant as they provided information related to the emergence of CRPA in new geographical locations.

We excluded studies with isolates of CRPA from environmental or animal samples, non-CRPA, other *Pseudomonas* spp., and studies solely reporting *P. aeruginosa* without KPC. Reviews (systematic, meta-analysis, and narrative), editorials, conferences, meeting abstracts, and duplicate reports were excluded. Articles in languages other than English and Spanish, with missing information and without full text, were not considered. Supplementary material (Table S3) shows the list of excluded studies with reasons for exclusion.

The quality of the studies was not considered an exclusion criterion for this systematic review.

### 4.3. Data Extraction and Analysis

The titles and abstracts of the literature search results were screened for eligibility and annotated in our database by one study researcher (D.F-H) following the PICO-based, predefined selection criteria.

The following data were extracted from each study according to inclusion criteria: (i) study-related variables (the first author’s name, year of publication, and country where the study was conducted), (ii) isolate-related variables (collection date, strain name, KPC variant, and STs), and (iii) genetic location (plasmid or chromosome) and genetic structures surrounding *bla*_KPC_ (transposons, NTE_KPC_, and/or insertion sequences). Information not provided by the article was classified as “not specified”. We performed typing of the plasmids that had an available accession number and did not report an incompatibility group using PlasmidFinder [87]. Additionally, an SLV analysis was conducted using PubMLST and eBURST tools [80,88]. The statistical analysis of the data obtained in this systematic review was performed using the package SPSS^®^. Finally, we provided a narrative synthesis of the results from the studies included, structured around the geographic distribution of *bla*_KPC_ reports and possible mobilization platforms.

### 4.4. Exploration of the bla_KPC_ Genetic Environment for P. aeruginosa in GenBank

A database was created for compiling information on the *bla*_KPC_ genetic environments in *P. aeruginosa* collected in GenBank (up to June 2022). All partial or fully sequenced nucleotide entries with more than 3000 bp upstream *bla*_KPC_ were included. General information of the entries, such as country, length, replicon type (linear or circular), *bla*_KPC_ variant, position in the genome, isolate name, and access information (GenBank and PMCID access numbers) were also registered. The nucleotide sequence for all entries was exported and compared against reference sequences of the NTE_KPC_ different subgroups (I, II, and III) previously described, whose classification criteria are based on the region upstream of *bla*_KPC_, as described by Chen et al. in 2014 [24]. The criteria to classify NTE_KPC_-I was the presence of ISK*pn27* or the characteristic *tnpR* of this subtype of elements; for NTE_KPC_-II, the insertion of Δ*bla*_TEM_; and for NTE_KPC_-III, the insertion of Tn*5563*/IS*6100* [24].

In the case of no association with previously reported genetic environments, the entry was characterized by manual curation using Artemis Comparison Tool (ACT), BLASTn and BLASTp [89,90], and specialized databases for MGE (TnRegistry and ISFinder) and resistance genes (CARD) [91,92]. Paired alignments were developed and plotted using Easyfig [93], showing identity between pairs in a window of 300 bp; the isolates in figures were organized according to the percentage of identity obtained from previously paired alignments of all the sequences in BLASTn.

### 4.5. Participation of Patients in the Study

The patients mentioned in this study were not involved in conducting or reporting plans for this systematic review.

## Figures and Tables

**Table 2:** *Pseudomonas aeruginosa* plasmids carrying *bla*_KPC_ completely sequenced and reported in the literature.

First Author	Year	Country	Strain	KPC Variant	ST	Plasmid Name	Length (bp)	Inc Group	Access Number	Ref
Naas	2013	Colombia	COL-1	KPC-2	ST308	pCOL-1	31,529	IncP-6	KC609323	[27]
Naas	2013	Colombia	PA-2	KPC-2	ST1006	pPA-2	7995	IncU	KC609322	[27]
Dai	2016	China	10265	KPC-2	NS	p10265-KPC	38,939	IncP-6	KU578314	[17]
Galetti	2016	Brazil	BH6	KPC-2	ST244	pBH6	3652	UI	LGVH01000782.1	[44]
Shi	2018	China	14057	KPC-2	NS	p14057A	51,663	UI	KY296095	[34]
Galetti	2019	Brazil	BH9	KPC-2	ST381	pBH6::Phage BH9	41,024	UI	CP029714	[46]
Hu	2019	China	PA1011	KPC-2	ST463	pPA1011	62.793	UI	MH734334	[35]
Li	2020	China	NK546	KPC-2	ST664	pNK546a	475,027	IncP-3-like (IncA/C)	MN433457	[19]
Wang	2021	China	Guangzhou-PaeC79	KPC-2	NS	pPAEC79	40,180	IncP-6	CP040685.1	[38]
Tartari	2021	Brazil	MIMA_PA2.1	KPC-2	ST312	pMIMA_PA2.1	7975	IncU	MT683857	[49]
Cai	2021	China	P23	KPC-2	ST463	pP23-KPC	40,937	UI	CP065418	[14]
Cai	2021	China	P33	KPC-2	ST463	pP33-2	48,306	UI	CP065414.1	[14]
Wozniak	2021	Chile	Pae-13	KPC-2	ST654	pPae-13	35,034	UI	MT949191	[60]
Yuan	2021	China	R31	KPC-2	NS	pR31-KPC	29,402	UI	CP061851	[39]
Zhu	2021	China	FAHZU31	KPC-2	ST244	pFAHZU31-KPC	24,350	UI	CP078010	[40]
Zhu	2021	China	FAHZU40	KPC-2	ST234	pFAHZU40-KPC	28,700	UI	CP078008	[40]
Zhu	2021	China	QZPH41	KPC-2	NS	pQZPH41-KPC	88,210	UI	CP064400	[40]
Zhu	2021	China	WTJH12	KPC-2	ST485	pWTJH12-KPC	396,963	UI	CP064404	[40]
Zhu	2021	China	ZPPH1	KPC-2	ST1212	pZPPH1-KPC	52,415	UI	CP077990	[40]
Cejas	2022	Argentina	PA_2047	KPC-2	ST654	pPA_2047	43,660	UI	MN082782	[62]
Cejas	2022	Argentina	PA_HdC	KPC-2	ST235	pPA_HdC	42,750	UI	OL780449	[62]
Tu	2022	China	PA2207	KPC-90	ST463	pPA2207_2	41,938	UI	CP080290	[41]
Li	2022	NS	NDTH10366	KPC-2	ST463	pNDTH10366-KPC	392,244	UI	CP064402	[70]
Silveira	2022	Brazil	CCBH28525	KPC-2	ST277	pCCBH28525	60,312	IncP	CP086065	[50]

Abbreviations: UI, incompatibility not associated with any existing Inc. group; NS, information not specified in the original article.

## Data Availability

Data relevant to the study are included in the manuscript or uploaded as Supplementary Information.

## Supplementary Materials

The following supporting information can be downloaded at: https://www.mdpi.com/article/10.3390/antibiotics12040658/s1, Table S1: Isolates that carry a novel NTE_KPC_; Table S2: Advanced search strategy for PubMed and Embase databases; and Table S3: Excluded studies with reason for exclusion.
