# Supplementary material for: Worldwide Dissemination of blaKPC Gene by Novel Mobilization Platforms in Pseudomonas aeruginosa: A Systematic Review
Source: Antibiotics (Basel). 2023 Mar 28;12(4):658. doi: 10.3390/antibiotics12040658 (PMC10134989; doi:10.3390/antibiotics12040658)
Supplement: Supplementary file 1 [file antibiotics-12-00658-s001.zip › antibiotics-2257061-supplementary.pdf]

## Supplementary Materials

**Supplementary Material S1.** Isolates that carry a novel $NTE_{KPC}$.

| $NTE_{KPC}$ subtype | Genome location | Strain | Plasmid name | Access number | Length (bp) | Country | Number of *bla*$_{KPC}$ copies | MLST |
|---|---|---|---|---|---|---|---|---|
| $NTE_{KPC}$-I | Plasmid | R31 | pR31-KPC | CP061851.1 | 29,402 | China | Single copy | NR |
| | Plasmid | PA2207 | unnamed | CP080290 | 41,938 | China | Single copy | ST463 |
| | Plasmid | BH6 | pBH6 | CM003767 | 3,652 | Brazil | Single copy | NR |
| | Plasmid | Not reported | YLH6_p3 | MK882885.1 | 49,162 | China | Double copy | NR |
| | Plasmid | P33 | pP33-2 | CP065414.1 | 48,306 | China | Single copy | NR |
| | Plasmid | C79 | p1 | CP040685 | 40,180 | China | Single copy | NR |
| | Plasmid | ZYPA | pZYPA01 | MZ050803.1 | 41,104 | China | Single copy | NR |
| | Chromosome | NDTH7329 | - | CP078006.1 | 7,144,260 | China | Six copies | ST463 |
| | Plasmid | QZPH16 | pQZPH16-KPC | CP078005.1 | 64,631 | China | Single copy | ST463 |
| | Plasmid | QZPH21 | pQZPH21-KPC | CP078003.1 | 58,802 | China | Single copy | ST463 |
| | Plasmid | SRRSH1120 | pSRRSH1120-KPC | CP078000.1 | 106,679 | China | Single copy | ST463 |
| | Chromosome | SRRSH1521 | - | CP077997.1 | 6,594,602 | China | Single copy | ST244 |
| | Plasmid | SRRSH2790 | pSRRSH2790-KPC | CP077995.1 | 62,848 | China | Single copy | ST463 |
| | Plasmid | NDTH10366 | pNDTH10366-KPC | CP064402.1 | 392,244 | China | Double copy | NR |
| | Chromosome | NDTH10366 | - | CP064401.1 | 6,974,425 | China | Three copies | ST463 |
| | Plasmid | SRRSH1408 | pSRRSH1408-KPC | CP064396.1 | 44,426 | China | Single copy | ST463 |
| | Plasmid | 1011 | p1011-KPC2 | MH734334.1 | 62,793 | China | Single copy | ST463 |
| $NTE_{KPC}$-II | Plasmid | 10265 | p10265-KPC | KU578314.1 | 38,939 | China | Single copy | NR |
| | Plasmid | HdC | unnamed | OL780449.1 | 42,750 | Argentina | Single copy | NR |
| | Plasmid | FAHZU40 | pFAHZU40-KPC | CP078008.1 | 28,700 | China | Single copy | ST234 |
| | Plasmid | SE5419 | pSE5419-3 | CP081349.1 | 42,487 | China | Double copy | ST697 |
| | Plasmid | SE5416 | pSE5416-KPC | MN894887.1 | 510,711 | China | Single copy | ST697 |
| | Plasmid | PA-2 | pPA-2 | KC609322.1 | 7,995 | Colombia | Single copy | ST1006 |

| | | | | | | | | |
|---|---|---|---|---|---|---|---|---|
| $NTE_{KPC}$-III | Plasmid | D5170990 | pD5170990 | KX169264.1 | 32,424 | Brazil | Single copy | NR |
| $NTE_{KPC}$-IV | Plasmid | CCBH28525 | pCCBH28525_KPC | CP086065.1 | 60,312 | Brazil | Double copy | NR |

**Abbreviations:** MLST, Multi-Locus Sequence Type; NR, Information not founded in the NCBI report nor via PubMLST.

**Supplementary Material S2.** Advanced search strategy for PubMed and Embase databases

| PUBMED | | |
|---|---|---|
| *Search* | *Query* | *Items found* |
| #1 | (((((Pseudomonas aeruginosa) OR (Bacterium aeruginosum)) OR (Bacillus aeruginosus)) | 51,030 |
| #2 | ((((((((((((((Carbapenems) OR (carbapenem)) OR (carbapenem antibiotics)) OR (antibiotics, carbapenem)) OR (doripenem)) OR (ertapenem)) OR (invanoz)) OR (invanz)) OR (ertapenem sodium)) OR (imipenem)) OR (imipemide)) OR (meropenem)) OR (merrem)) OR (ronem)) OR (penem))) | 27,454 |
| #3 | ((((beta-lactamase KPC-2) OR (beta-lactamase KPC-3)) OR (blaKPC)) OR (bla KPC))) **AND** ((((((((((dissemination) OR (transposon resolvases)) OR (transposon)) OR (DNA transposable elements)) OR (integrons)) OR (integron)) OR (plasmids)) OR (plasmid)) OR (episomes)) OR (episome)) | 1,115 |
| **Final search (#1 AND #2 AND #3)** | (((((Pseudomonas aeruginosa) OR (Bacterium aeruginosum)) OR (Bacillus aeruginosus)) **AND** (((((((((((((((Carbapenems) OR (carbapenem)) OR (carbapenem antibiotics)) OR (antibiotics, carbapenem)) OR (doripenem)) OR (ertapenem)) OR (invanoz)) OR (invanz)) OR (ertapenem sodium)) OR (imipenem)) OR (imipemide)) OR (meropenem)) OR (merrem)) OR (ronem)) OR (penem))) **AND** ((((beta-lactamase KPC-2) OR (beta-lactamase KPC-3)) OR (blaKPC)) OR (bla KPC))) **AND** ((((((((((dissemination) OR (transposon resolvases)) OR (transposon)) OR (DNA transposable elements)) OR (integrons)) OR (integron)) OR (plasmids)) OR (plasmid)) OR (episomes)) OR (episome)) | 65 |

| EMBASE | | |
|---|---|---|
| *Search* | *Query* | *Items found* |
| #1 | 'pseudomonas aeruginosa'/exp/ | 93,588 |
| #2 | ('carbapenem'/exp OR 'carbapenem' OR 'doripenem'/exp OR 'doribax' OR 'doripenem' OR 'finibax' OR 'ertapenem'/exp OR 'ertapenem' OR 'ertapenem sodium' OR 'invanoz' OR 'invanz' OR 'imipenem'/exp OR 'formiminothienamycin' OR 'imipemide' OR 'imipenem' OR 'meropenem'/exp OR 'mepem' OR 'meronem' OR 'meropen' OR 'meropenem' OR 'merrem' OR 'merrem i.v.') | 87,342 |
| #3 | ('blakpc gene'/exp OR 'blakpc 2 gene'/exp OR 'blakpc 3 gene'/exp OR kpc) AND ('transposon'/exp OR 'tes (transposable elements)' OR 'jumping gene' OR 'transposable element' OR 'transposable elements' OR 'transposon' OR 'dissemination'/exp OR 'integron'/exp OR 'plasmid'/exp OR 'bacterial plasmid' OR 'gene, plasmid' OR 'plasmid' OR nte OR 'gene cassette'/exp) | 1,255 |
| **Final search (#1 AND #2 AND #3)** | 'pseudomonas aeruginosa'/exp **AND** ('carbapenem'/exp OR 'carbapenem' OR 'doripenem'/exp OR 'doribax' OR 'doripenem' OR 'finibax' OR 'ertapenem'/exp OR 'ertapenem' OR 'ertapenem sodium' OR 'invanoz' OR | 113 |

| | |
|---|---|
| | 'invanz' OR 'imipenem'/exp OR 'formiminothienamycin' OR 'imipemide' OR 'imipenem' OR 'meropenem'/exp OR 'mepem' OR 'meronem' OR 'meropen' OR 'meropenem' OR 'merrem' OR 'merrem i.v.') **AND** ('blakpc gene'/exp OR 'blakpc 2 gene'/exp OR 'blakpc 3 gene'/exp OR kpc) **AND** ('transposon'/exp OR 'tes (transposable elements)' OR 'jumping gene' OR 'transposable element' OR 'transposable elements' OR 'transposon' OR 'dissemination'/exp OR 'integron'/exp OR 'plasmid'/exp OR 'bacterial plasmid' OR 'gene, plasmid' OR 'plasmid' OR nte OR 'gene cassette'/exp) |

**Supplementary Material S3.** Excluded studies with reason for exclusion.

| First author | Year | Reason for exclusion |
|---|---|---|
| Nordmann | 2002 | Review article |
| Poirel | 2002 | Isolates of *Pseudomonas aeruginosa* without KPC |
| Poirel | 2002 | Isolates of *Pseudomonas aeruginosa* without KPC |
| Bedenić | 2005 | Review article |
| Livermore | 2006 | Review article |
| LaBombardi | 2007 | Review article |
| Poirel | 2007 | Review article |
| Pitout | 2008 | Review article |
| Goldfarb | 2009 | Irrelevant pathogen |
| Pfeifer | 2009 | Non-relevant studies |
| Walsh | 2009 | Review article |
| Pfeifer | 2010 | Review article |
| Cuzon | 2010 | Review article |
| AmbrožičAvguštin | 2010 | Isolates of *Pseudomonas aeruginosa* without KPC |
| Walsh | 2010 | Review article |
| Nordmann | 2010 | Review article |
| Nath | 2010 | Non-relevant studies |
| Tian | 2011 | Isolates of *Pseudomonas aeruginosa* without KPC |
| Hrabák | 2011 | Irrelevant pathogen |
| Castanheira | 2011 | Isolates of *Pseudomonas aeruginosa* without KPC |
| Richter | 2011 | Isolates of *Pseudomonas aeruginosa* without KPC |
| Naas | 2011 | Isolates of *Pseudomonas aeruginosa* without KPC |
| Jánvári | 2011 | Irrelevant pathogen |
| Drieux | 2011 | Isolates of *Pseudomonas aeruginosa* without KPC |
| Mataseje | 2012 | Isolates of *Pseudomonas aeruginosa* without KPC |
| Hong | 2012 | Isolates of *Pseudomonas aeruginosa* without KPC |
| Castanheira | 2012 | Isolates of *Pseudomonas aeruginosa* without KPC |
| Damjanova | 2012 | Irrelevant pathogen |

| | | |
|---|---|---|
| Castanheira | 2012 | Isolates of *Pseudomonas aeruginosa* without KPC |
| Bryant | 2013 | Isolates of *Pseudomonas aeruginosa* without KPC |
| Kaase | 2013 | Isolates of *Pseudomonas aeruginosa* without KPC |
| Chen | 2013 | Irrelevant pathogen |
| Liu | 2014 | Language |
| Martínez | 2014 | Irrelevant pathogen |
| Zheng | 2015 | Isolates of *Pseudomonas aeruginosa* without KPC |
| ND | 2015 | Review article |
| Khatun | 2015 | Isolates of *Pseudomonas aeruginosa* without KPC |
| Ruppé | 2015 | Review article |
| Sun | 2016 | Isolates of *Pseudomonas aeruginosa* without KPC |
| Vanegas | 2016 | Isolates of *Pseudomonas aeruginosa* without KPC |
| Martinez | 2016 | Irrelevant pathogen |
| Nachimuthu | 2016 | Isolates of *Pseudomonas aeruginosa* without KPC |
| Rolain | 2016 | Review article |
| Ng | 2017 | Non-human study |
| Manohar | 2017 | Isolates of *Pseudomonas aeruginosa* without KPC |
| Beyrouthy | 2017 | Irrelevant pathogen |
| ALI | 2017 | Non-human study |
| Nascimento | 2017 | Non-human study |
| Manohar | 2017 | Isolates of *Pseudomonas aeruginosa* without KPC |
| Bloemberg | 2017 | Non-relevant studies |
| Bush | 2017 | Review article |
| Kocsis B | 2017 | Review article |
| Mathlouthi | 2017 | Review article |
| Lamba | 2017 | Non-human study |
| Khurana | 2017 | Isolates of *Pseudomonas aeruginosa* without KPC |
| Jaillard | 2017 | Non-relevant studies |
| Johnning | 2018 | Isolates of *Pseudomonas aeruginosa* without KPC |
| Zowawi | 2018 | Isolates of *Pseudomonas aeruginosa* without KPC |
| Castanheira | 2018 | Non-relevant studies |
| Castanheira | 2018 | Isolates of *Pseudomonas aeruginosa* without KPC |
| Singh-Moodley | 2018 | Isolates of *Pseudomonas aeruginosa* without KPC |
| Wen | 2018 | Isolates of *Pseudomonas aeruginosa* without KPC |
| Karampatakis | 2018 | Review article |
| Alves | 2018 | Irrelevant pathogen |
| Yoon | 2018 | Irrelevant pathogen |
| Wyres | 2018 | Irrelevant pathogen |
| Pournajaf | 2018 | Non-relevant studies |

| | | |
|---|---|---|
| Gomez-Gamboa | 2019 | Isolates of *Pseudomonas aeruginosa* without KPC |
| Mangat | 2019 | Non-relevant studies |
| Lee | 2019 | Isolates of *Pseudomonas aeruginosa* without KPC |
| Ortiz de la Rosa | 2019 | Non-relevant studies |
| Zhu | 2019 | Isolates of *Pseudomonas aeruginosa* without KPC |
| Song | 2019 | Isolates of *Pseudomonas aeruginosa* without KPC |
| O'Neall | 2020 | Isolates of *Pseudomonas aeruginosa* without KPC |
| Silva Júnior | 2020 | Isolates of *Pseudomonas aeruginosa* without KPC |
| Beirão | 2020 | Non-relevant studies |
| Botelho | 2020 | Non-relevant studies |
| Lima | 2020 | Irrelevant pathogen |
| Nishida | 2020 | Irrelevant pathogen |
| AlAmri | 2020 | Irrelevant pathogen |
| Silva | 2020 | Isolates of *Pseudomonas aeruginosa* without KPC |
| Bush | 2020 | Review article |
| Halat | 2020 | Review article |
| Pathak | 2020 | Isolates of *Pseudomonas aeruginosa* without KPC |
| Ramirez | 2020 | Isolates of *Pseudomonas aeruginosa* without KPC |
| Janse | 2021 | Non-relevant studies |
| Yan | 2021 | Irrelevant pathogen |
| Yee | 2021 | Isolates of *Pseudomonas aeruginosa* without KPC |
| Majlander | 2021 | Non-human study |
| Al-Zahrani | 2021 | Isolates of *Pseudomonas aeruginosa* without KPC |
| Nafplioti | 2021 | Isolates of *Pseudomonas aeruginosa* without KPC |
| Sanou | 2021 | Irrelevant pathogen |
| Borelli | 2021 | Isolates of *Pseudomonas aeruginosa* without KPC |
| Dos Santos | 2021 | Non-human study |
| Janssen | 2021 | Non-human study |
| Huang | 2021 | Irrelevant pathogen |
| Nirwan | 2021 | Irrelevant pathogen |
| Jean | 2022 | Review article |
| Ayoub Moubareck | 2022 | Review article |
